# Interaction between microorganisms and flavour products during cigar fermentation promoted by citrus Reticulata-“Chenpi” derived *Enterobacter* G5Z-2: based on multi-omics studies and microbial profiles

**DOI:** 10.3389/fbioe.2026.1785975

**Published:** 2026-05-08

**Authors:** Jian Zhang, Chong Chen, Yang Hu, Shiru Jia, Bo Li, Wanrong Hu, Yun Jia, Dongliang Li, Yuanfa Liu

**Affiliations:** 1 State Key Laboratory of Food Nutrition and Safety, College of Biotechnology, Tianjin University of Science and Technology, Tianjin, China; 2 Future Food (Baima) Research Institute, Nanjing, China; 3 State Key Laboratory of Food Science and Technology, School of Food Science and Technology, National Engineering Research Center for Functional Food, National Engineering Laboratory for Cereal Fermentation Technology, Collaborative Innovation Center of Food Safety and Quality Control in Jiangsu Province, Jiangnan University, Wuxi, China; 4 Laboratory of Industrial Fermentation Microbiology (Ministry of Education), Tianjin University of Science and Technology, Tianjin, China; 5 School of Grain Science and Technology, Jiangsu University of Science and Technology, Zhenjiang, China; 6 China Tobacco Technology Innovation Center for Cigar, China Tobacco Sichuan Industrial Co. Ltd, Chengdu, China

**Keywords:** chenpi, cigar, cigar flavour, *Enterobacter*, fermentation

## Abstract

**Introduction:**

Cigar fermentation is crucial for developing its characteristic aroma, exogenous microorganisms can be used to enhance fermentation. It is reported that the citrus reticulata ‘Chachi’ (Chenpi, a traditional fermented ingredient) extract can improve the flavor of cigarette. However, there is no report on the influence of Chenpi-derived microorganisms on the fermentation process and flavor quality of cigar tobacco leaves (CTLs) till now.

**Methods:**

A fermentation strain (*Enterobacter hoffmannii*, G5Z-2) was isolated from Chenpi, and it was applied as a bioaugmentation agent in CTLs fermentation. A multi-omics approach, including metagenomics and metabolomics, was employed to investigate its impact.

**Results:**

Inoculation with G5Z-2 significantly altered the microbial community structure, suppressing native *Pseudomonas* and reducing overall alpha diversity while enriching beneficial genera like *Aspergillus* and *Staphylococcus*. Metabolomic analysis revealed substantial restructuring of metabolic pathways, particularly the enrichment of amino acid metabolism (such as arginine biosynthesis and phenylalanine metabolism) and nicotinate/nicotinamide metabolism. This led to accelerated degradation of proteins and amino acids, providing precursors for Maillard reaction, and a marked increase (57.5%) in total volatile flavour compounds, including key aroma constituents from carotenoid and cembranoid degradation.

**Conclusion:**

The Chenpi-derived *E. hoffmannii* G5Z-2 optimises the fermentation process by modulating the microbial consortium and driving metabolic shifts towards favourable flavour development, demonstrating significant potential for improving the quality of Chinese-style cigars.

## 1 Introduction

Tobacco is considered as a major global commercial crop, and it is cultivated in various countries, including China, the USA, and Brazil (Yang H et al., 2025). Cigars are products formed through processes such as air-curing, fermentation, and rolling, possessing a distinctive aroma and unique taste. Among these processes, fermentation is the core process determining cigar quality (Su et al., 2024). The fermentation and ageing process of cigar tobacco leaves (CTLs) is a natural fermentation process. Accompanied with slow, long-term microbial fermentation, it imparts a pleasant aroma and forms the unique cigar flavour profile (Allem et al., 2019). Consequently, compared with flue-cured tobacco, cigars exhibit a more mellow and rich flavour, often containing notes of fruit, nuts, coffee, milk, and cedar (Morris and Fiala, 2015). However, a significant gap remains between the quality of Chinese tobacco and internationally recognised high-quality tobacco (Yin et al., 2025). CTLs contain substantial amounts of starch, protein, and macromolecules, which can adversely affect the combustibility and odour of the tobacco. The degradation of starch and protein into small molecules can significantly enhance the formation of aromatic compounds and improve leaf quality (Zhang et al., 2024).

Cigar fermentation is a complex solid-state fermentation system, essentially involving a combination of physical, chemical, and biological processes (Wu et al., 2023). It is influenced by various factors. The metabolic activities of the cigar microbial community significantly contribute to the formation of the cigar aroma (Banožić et al., 2020). Intervention with exogenous microorganisms during the industrial fermentation stage of cigars is a relatively effective strategy. Current research focuses on eluting, directionally enriching, and isolating cultivable microorganisms from the CTLs. Adding exogenous microorganisms is an effective way to improve the quality of CTLs, such as *Acinetobacter*, *Candida* and *Bacillus altitudinis* (Jia et al., 2023; Song et al., 2024; Zheng et al., 2022a). A novel microbial fermentation medium isolated from an edible medicinal fungus, *Tremella aurantialba SCT-F3* can improve the sensory quality of cigar filler leaves (Zhang et al., 2023).

Native Americans have artificially intervened and adjusted tobacco leaf fragrance by adding citrus peels (Zheng et al., 2022b). Chenpi is a Chinese medicinal and edible substance with unique flavour characteristics, which is dried tangerine peel of *Citrus reticulata*, renowned for its “sweet, aromatic, and mellow” properties. It primarily contains active components such as volatile oils, flavonoids, alkaloids (Yi et al., 2015). Researches indicated that the quality of Chenpi may be influenced its core microorganism community (Li et al., 2025; Huang et al., 2024). Chenpi is an ideal candidate for developing specialised fermentation media and enhancing the quality of Chinese-style cigars. Research showed that chenpi extract can increase the content of citric acid, improve the acid-base balance of smoke, and significantly enhance the fruity and woody aroma characteristics of smoke (Hu et al., 2024). However, there is no research on using Chenpi-derived microorganisms for cigar fermentation.

The main objectives of the present research were (1) screen *Enterobacter* strain from Chenpi as a CTLs bioaugmentation agent; (2) investigated the dynamic changes in the microbial community within the fermentation system, analyse physicochemical indicators and metabolites affecting cigar quality with metabolomics; (3) explore the interactions between microorganisms in the cigar fermentation system and their correlations with key metabolites.

## 2 Materials and methods

### 2.1 Strain screening

Directed isolation and screening of microorganisms from Chenpi. A full-nutrient agar medium was used as the base, supplemented with 1% (w/v) powdered 80-mesh CTLs before sterilisation. PBS buffer was used to wash the surface microorganisms from 1-year, 3-year, 5-year, and 10-year Chenpi. The samples were shaken on a shaker for 30 min, and the eluate was then spread onto plates for the directed screening of cultivable microorganisms. Twelve Chenpi-derived microbial strains were isolated. These strains were capable of growing on media containing 1% CTLs and were preserved in the Cell Laboratory of Future Food (Baima) Research Institute. The twelve microbial strains were subjected to expanded culture and centrifugation to collect cell pellets. These were added to CTLs at 2‰ (m/v) for fermentation experiments.

### 2.2 Strain isolation and purification

For thorough elution, 1 g of CTLs sample was aseptically added to 9 mL of sterile phosphate-buffered saline (PBS, 0.1 M, pH 7.2) and shook with a speed of 180 rpm at 30 °C for 1 h. 1 mL of the eluate was taken for serial 10-fold gradient dilution. 100 μL of the diluted solution was aspirated and evenly spread onto LB agar plates. The plates were inverted and incubated at 30 °C for 24 h. After single colonies appeared, they were preliminarily distinguished based on colony morphology, color and size. Single colonies were picked and repeatedly purified with the streak plate method until pure cultures were obtained. One strain, designated G5Z-2, was identified as *Enterobacter* by 16S rRNA gene sequence analysis and preserved in the laboratory microbial strain collection for subsequent fermentation experiments. The strain has been deposited with the China General Microbiological Culture Collection Center (CGMCC) under accession number No. 33624.

### 2.3 CTLs fermentation

The initial moisture content of the CTLs was determined. The amount of water required for conditioning was calculated using the weight-based rehydration method to bring the leaf moisture content to 28%. After moisture equilibration, the leaves were placed at 35 °C and 75% humidity for fermentation. The required amount of bacterial cells was calculated based on an inoculation rate of 2‰. The secondary seed culture of G5Z-2 was centrifuged at 10,000 r/min for 5 min, the supernatant was discarded, and the cell pellet was collected. The cell pellet was washed three times with sterile distilled water to remove the medium, finally resuspended in sterile distilled water. The resulting bacterial suspension was evenly sprayed onto the surface of the CTLs. All fermentation experiments were performed with at least three independent biological replicates to ensure statistical reliability. The fermentation period was 30 days. The fermentation experiment in this study comprised three distinct groups: CTLs with no fermentation process (the unfermented group), CTLs fermented with ultrapure water (the fermented-control group) and CTLs fermented with equivalent volume of G5Z-2 bacterial suspension (the fermented-G5Z-2 group).

### 2.4 Main chemical components analysis

The content of total sugars and reducing sugars was determined according to the tobacco industry standard “Tobacco and tobacco products—Determination of water-soluble sugars—Continuous flow method” (YC/T 159–2019). Total nitrogen content was determined according to “Tobacco and tobacco products—Determination of total nitrogen—Continuous flow method” (YC/T 161–2002). Free amino acid content was determined according to “Determination of free amino acids in plants” (GB/T 30,987-2020).

### 2.5 Enzyme activity analysis

Dry weight CTLs powder acquisition: 500 g of CTLs was transferred to a mortar and ground continuously with liquid nitrogen until complete tissue disruption was achieved. The resulting coarse powder was subsequently processed with a high-speed grinder (Model FW80, China) for secondary pulverization, followed by sieving through a 60-mesh sieve to obtain the final dry CTLs powder.

Crude enzyme extraction: 2.5 g of CTLs powder was added to 30 mL of PBS and oscillated for extraction at room temperature for 1 h. It was then centrifuged at 7,000 r/min for 15 min, then the supernatant was separated to obtain the crude enzyme extract. The enzyme activity was calculated as units per Gram of dry weight (DW) CTLs powder (U/g).

Amylase activity (AL): The method of Keharom was used with modifications (Keharom et al., 2016). The change in absorbance of the system was detected at a wavelength of 660 nm to calculate enzyme activity. Amylase activity was defined as the amount of crude enzyme that hydrolyses 1 mg of soluble starch per minute under conditions of 60 °C and pH 5.6, defined as one unit of enzyme activity (U).

Lipoxygenase activity (LPO): A spectrophotometric continuous assay method was used (Gökmen et al., 2002), dynamically monitoring the change in absorbance of the reaction system at a wavelength of 234 nm. Lipoxygenase activity was defined as the change in absorbance of the reaction system at 234 nm by 0.001 per minute under conditions of 30 °C and pH 6.0, defined as one unit of enzyme activity (U).

Polyphenol oxidase activity (PPO) was assayed according to “Determination of polyphenol oxidase activity in wheat flour” (LS/T 6124-2017). Peroxidase activity (POD) was assayed according to “Method for determination of horseradish peroxidase activity” (GB/T 32,131-2015). Protease activity (PRO) was assayed according to “Quality requirements for enzyme preparations” (GB/T 23,527.1-2023).

### 2.6 Volatile aroma compounds analysis

Sample preparation: 0.5 g of CTLs powder was weighed into a 20 mL headspace vial, and 30 μL of 1 mg/mL phenethyl acetate (internal standard) was added.

Gas chromatography conditions: DB-5MS capillary column (60 m × 0.25 mm × 1.0 μm); the temperature program was set as follows: initial temperature 60 °C, then increased to 250 °C at 2 °C/min, then increased to 290 °C at 5 °C/min; injector temperature 290 °C; split ratio 10:1; carrier gas helium (He), flow rate 1.5 mL/min.

Mass spectrometry conditions: Electron ionisation (EI) source; ion source temperature 230 °C; electron energy 70 eV; quadrupole temperature 150 °C; mass scan range 26–400 amu; monitoring mode: full scan.

Qualitative and quantitative analysis: Compounds were identified by matching mass spectra with the National Institute of Standards and Technology (NIST 20) and Wiley mass spectral databases. Quantification was performed using the internal standard method.

### 2.7 Microbial diversity analysis

Acording to the preliminary sensory evaluation results, samples fermented for 1, 14, and 30 days was used for microbial diversity analysis. Microbiome total DNA extraction and detection: Nucleic acids were extracted using the OMEGA Soil DNA Kit, D5625-01, (Omega Bio-Tek, Norcross, GA, USA). DNA was quantified using a UV spectrophotometer. PCR amplification targeted the hypervariable V3-V4 region (∼468 bp) of the bacterial 16S rRNA gene using specific primers: 338F (5′-barcode + ACT​CCT​ACG​GGA​GGC​AGC​A-3′) and 806R (5′-GGACTACHVGGGTWTCTAAT-3′). Library construction: Libraries were constructed using Illumina’s TruSeq Nano DNA LT Library Prep Kit. Sequencing: Paired-end sequencing (2 × 250 bp) was performed on an Illumina NovaSeq platform using the NovaSeq 6000 SP Reagent Kit (500 cycles).

### 2.8 Metagenomic analysis

Metagenomic DNA extraction and shotgun sequencing were performed on CTL samples fermented for 14 days. Metagenomic DNA extraction and shotgun sequencing: Total microbial genomic DNA was extracted using the OMEGA DNA Kit (D5625-01) and stored at −20 °C. The quantity and quality of the extracted DNA were assessed using a NanoDrop ND-1000 spectrophotometer and agarose gel electrophoresis. The extracted microbial DNA was processed using the Illumina TruSeq Nano DNA LT Library Preparation Kit to construct metagenomic shotgun sequencing libraries with an insert size of 400 bp. Each library was sequenced on the Illumina HiSeq X Ten platform using a PE150 strategy by Personal Biotechnology Co., Ltd.

Metagenomic analysis: Based on the taxonomic and functional characteristics of non-redundant genes, LEfSe (Linear Discriminant Analysis Effect Size) was performed with default parameters to detect differentially abundant taxa and functions between different groups. Beta diversity analysis was conducted using the Bray-Curtis distance metric and visualised via Principal Coordinates Analysis (PCoA), Non-metric Multidimensional Scaling (NMDS), and Unweighted Pair Group Method with Arithmetic Mean (UPGMA) hierarchical clustering to investigate compositional and functional changes in the microbial communities among samples.

### 2.9 Metabolomic analysis

100 μg of CTLs sample was weighed into a 2 mL centrifuge tube. 500 μL of pre-cooled methanol:acetonitrile:water mixture (2:2:1, v/v/v, containing 5 ppm 2-chloro-L-phenylalanine as internal standard) was added, followed by two steel beads. The mixture was vortexed for 30 s, then placed in a high-throughput tissue grinder and ground at 55 Hz for 60 s; this step was repeated once. The sample was then sonicated in an ultrasonic cleaner for 10 min, placed in a −20 °C freezer for 30 min, and centrifuged at 12,000 rpm and 4 °C for 10 min. The supernatant was collected and filtered through a 0.22 μm membrane. The filtrate was transferred to an injection vial. 10 μL of the sample filtrates were pooled to create a quality control (QC) sample for evaluating instrument stability and data reliability.

Raw data were converted to mzXML format with ProteoWizard. Peak alignment, retention time correction, and peak area extraction were then performed with XCMS software. The data extracted by XCMS were first subjected to metabolite identification and data preprocessing, followed by experimental data quality assessment, and finally data analysis. This project utilised both a locally self-built database and public databases for library searching (Human Metabolome Database (HMDB) (http://www.hmdb.ca), Metlin (http://metlin.scripps.edu), MassBank (http://www.massbank.jp/), mzCloud (https://www.mzcloud.org)). Metabolites in the biological samples were structurally identified by matching their retention time, molecular mass (mass error <10 ppm), MS/MS fragmentation spectra, and collision energy with information in the databases. The identification results were strictly verified and confirmed. The identification level was Level 2 or higher.

### 2.10 Statistical analysis

SPSS 26 and Excel software were used for data analysis, and Origin 2021 software was used for plotting. SIMCA 14.1 was used for Partial Least Squares-Discriminant Analysis (PLS-DA). R language packages and TB tools were used for Principal Component Analysis (PCA), hierarchical clustering analysis, cluster heatmap generation, and Support Vector Machine (SVM) modelling.

KEGG annotation and enrichment analysis of differential metabolites were performed. Identified metabolites were annotated using the KEGG Compound database (http://www.kegg.jp/kegg/compound/). The annotated metabolites were then mapped to the KEGG Pathway database (http://www.kegg.jp/kegg/pathway.html). Pathways mapped by significantly regulated metabolites were input into MSEA (Metabolite Set Enrichment Analysis), and their significance was determined by the p-value from a hypergeometric test.

## 3 Results

### 3.1 Isolation and identification of G5Z-2

Following the procedure described in Section 2.1, twelve Chenpi-derived microbial strains were isolated. Based on preliminary sensory evaluation, a group of cigar tobacco leaves with the optimal flavor was selected (data not shown), and its strain was designated as G5Z-2 for subsequent experimental characterization.

After 12 h of cultivation on LB plates, the colony diameter of G5Z-2 was 1–2 mm. The colonies were circular with neat edges, opaque, greyish-white on the obverse, convex in the centre, with a bright, smooth surface, moist texture, and were easily picked. Gram staining was negative. 16S sequencing results indicated that G5Z-2 belonged to the genus *Enterobacter* (*Enterobacter* spp.).

**FIGURE 1:**
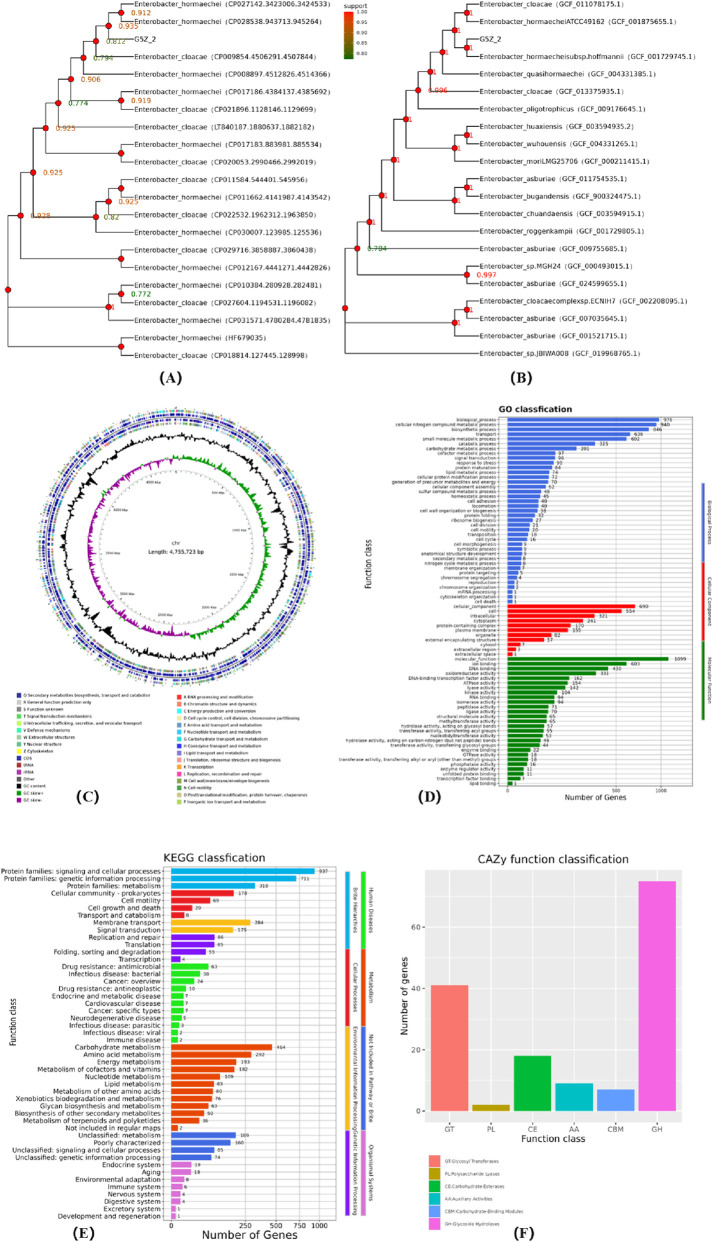
Sequencing, classification, and gene annotation of the G5Z-2 strain. (**(A)** 16S gene phylogenetic tree; **(B)** Core gene phylogenetic tree; **(C)** CGView genome circle map; **(D)** GO annotation of protein-coding genes; **(E)** KEGG statistics; **(F)** CAZy functional classification).

Figure 1 shows the whole genome sequencing results of G5Z-2, including gene function and metabolic predictions. The 16S and whole-genome family trees show that G5Z-2 has the closest genetic composition to *Enterobacter hormaechei subsp. hoffmannii* (Figures 1A,B). The chromosome size of G5Z-2 is 4.76 Mb with a GC content of 55.23% (Figure 1C). The strain contains three plasmids, among which plasmid2 shows homology with *Klebsiella* and plasmid3 with *Salmonella*. Figures 1D–F show the number of genes related to biological processes. The most abundant functional genes in the G5Z-2 genome were those for “cellular nitrogen compound metabolic process” and “biosynthetic process”, followed by genes involved in various components inside and outside the cell, such as cell, cytoplasm, and plasma membrane, as well as functional genes such as DNA-binding activity and ATPase activity. Furthermore, G5Z-2 carries an efficient carbohydrate metabolism system, including 75 glycoside hydrolases and 41 glycosyltransferases, demonstrating the potential to degrade biomass such as cellulose and starch (F). *Enterobacter* species have been reported for their discovery and application in metabolising macromolecular carbohydrates like cellulose (Bagewadi et al., 2020), and *Klebsiella* species are known for their role in degrading nicotine and lignin.

### 3.2 Chemical components and enzyme activity

**FIGURE 2:**
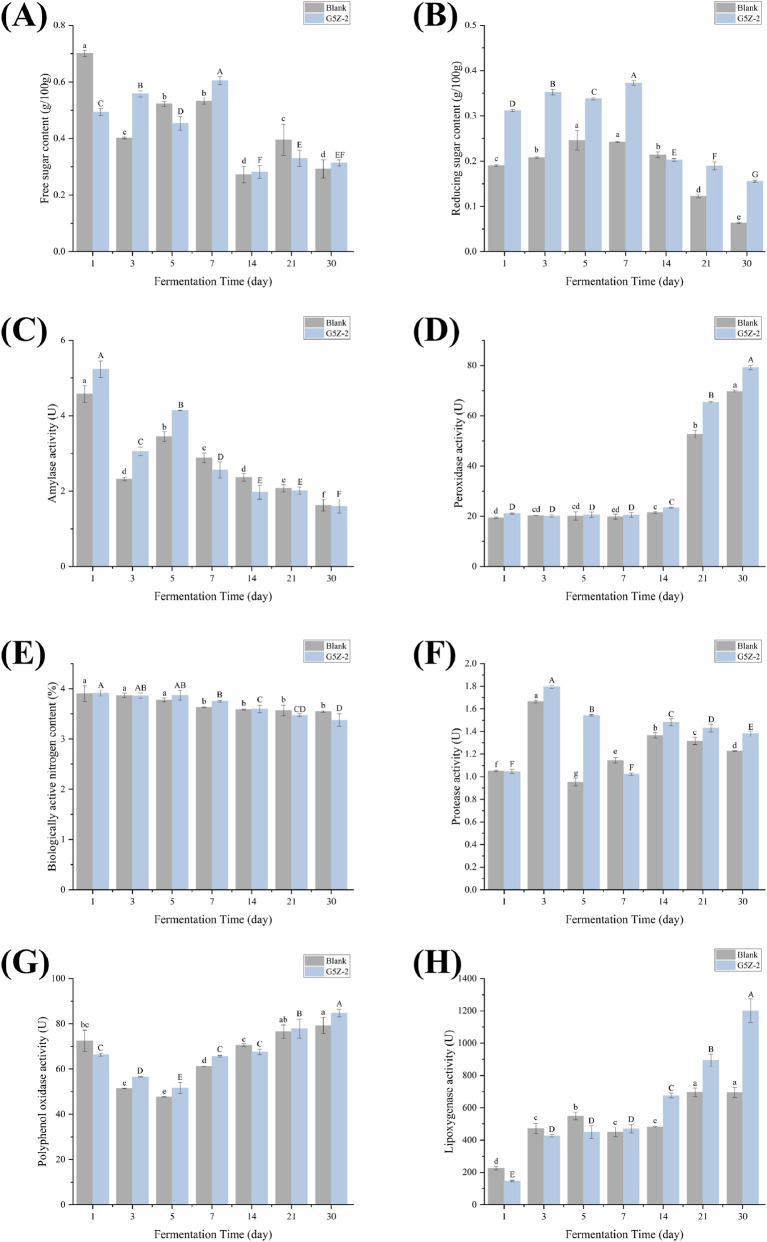
Changes of conventional chemical components and enzyme activities in CTLs during fermentation. **(A)** The free sugar content of the fermented-G5Z-2 and fermented-control group; **(B)** the reducing sugar content of the fermented-G5Z-2 and fermented-control group; **(C)** the amylase activity of the fermented-G5Z-2 and fermented-control group; **(D)** the peroxidase activity of the fermented-G5Z-2 and fermented-control group; **(E)** the bioavailable nitrogen content of the fermented-G5Z-2 and fermented-control group; **(F)** the protease activity of the fermented-G5Z-2 and fermented-control group; **(G)** the polyphenol oxidase activity of the fermented-G5Z-2 and fermented-control group; **(H)** the lipoxygenase activity of the fermented-G5Z-2 and fermented-control group).

CTLs were fermented for 30 days, and samples were taken on day 1, 3, 5, 7, 14, 21, and 30 to measure free sugar and reducing sugar content. Figure 2A shows that the free sugar content in the fermented-G5Z-2 group initially increased, peaking at 0.37 g/100 g on day 7, and then gradually decreased. The fermented-control group showed a similar trend overall, but the free sugar level at the end of fermentation was lower than that in the fermentation group. The results for reducing sugars (Figure 2B) show that during fermentation, reducing sugars first increased steadily and then decreased sharply. Throughout the entire fermentation period, the reducing sugar level in the fermented-control group was consistently significantly lower than the fermented-G5Z-2 group, with a difference exceeding 0.1 g/100 g. Figure 2C shows that amylase activity was highest on day 1 for both the fermented-control and fermented-G5Z-2 groups (4.58 ± 0.22 U and 5.23 ± 0.22 U, respectively), then slowly decreased, reaching the lowest point at the end of fermentation (1.63U ± 0.15 and 1.60U ± 0.18). However, a rebound was observed on day 5 (3.45 ± 0.13 U and 4.14 ± 0.004 U). Peroxidase activity results (Figure 2D) show that levels in both the fermented-control and fermented-G5Z-2 groups remained around 20 U for the first 14 days but increased sharply to a peak in the final 2 weeks. Ultimately, the fermented-G5Z-2 group had a significantly higher level (79.22 ± 0.87 U) compared to the fermented-control group (69.70 ± 0.52 U). Figure 2E shows that the bioavailable nitrogen level in both the fermented-control and fermented-G5Z-2 groups decreased steadily from 3.9% to 3.4% during fermentation, with no significant difference between the groups. Figure 2F shows that protease levels in both the fermented-control and fermented-G5Z-2 groups started at around 1.05 U on day 1, rapidly increased to above 1.6 U by day 3, and then continued to decrease throughout the subsequent fermentation period. A significant difference appeared on day 5, with the fermented-control group enzyme activity at 0.95 ± 0.03 U and the fermented-G5Z-2 group enzyme activity reaching 1.54 ± 0.01 U. Figure 2G shows that polyphenol oxidase activity in both the fermented-control and fermented-G5Z-2 groups was above 66 U on day 1, dropped to the lowest point on day 5 (47.76 ± 0.16 U and 51.58 ± 2.42 U, respectively), and reached a peak at the end of fermentation (79.27 ± 3.56 U and 84.73 ± 1.89 U, respectively). Figure 2H shows that lipoxygenase activity in both the fermented-control and fermented-G5Z-2 groups was lowest on day 1 and increased continuously as fermentation progressed. The fermented-control group increased from 225 ± 10.56 U to 694 ± 31.11 U, while the fermented-G5Z-2 group increased from 147 ± 5 U to 1201 ± 73.33 U.

### 3.3 Free amino acids

A total of 19 free amino acids were detected in the cigar samples (Table 1). As fermentation progressed, the level of bioavailable nitrogen gradually decreased in both the fermented-control and fermented-G5Z-2 groups. Table 1 show that the free amino acids content of the fermented-control and fermented-G5Z-2 groups were lower than the unfermented CTLs. The introduction of G5Z-2 fermentation directly led to greater consumption of most amino acids. Alanine, γ-aminobutyric acid, serine, valine, threonine, asparagine, aspartic acid, lysine, glutamic acid, phenylalanine, and arginine were all significantly lower than in the fermented-control group. Glycine was the only amino acid completely depleted due to the introduction of G5Z-2 fermentation. However, proline was an exception; although it decreased significantly after fermentation, it decreased more significantly in the fermented-control group compared to the fermented-G5Z-2 group.

**TABLE 1:** Free amino acid content in CTLs before and after fermentation (unit: µg/g).

Amino acid	Unfermented	Fermented-control	Fermented-G5Z-2	Metabolic direction
Glycine	14.28 ± 1.54^a^	9.00 ± 0.91^b^	0.00^c^	​
Alanine	24.06 ± 2.18^a^	15.98 ± 1.04^b^	8.55 ± 0.56^c^	​
γ-Aminobutyric acid	23.51 ± 1.97^a^	18.10 ± 1.27^b^	11.10 ± 0.35^c^	Converted to succinate (Yang H et al., 2025)
Serine	19.21 ± 1.78^a^	18.33 ± 0.78^a^	9.17 ± 0.17^b^	​
Proline	42.64 ± 4.53^a^	19.93 ± 1.16^c^	23.57 ± 1.03^b^	​
Valine	8.11 ± 0.91^a^	7.43 ± 0.42^a^	3.90 ± 0.57^b^	​
Threonine	29.34 ± 1.93^a^	19.60 ± 1.39^b^	12.91 ± 0.90^c^	Converted to α-ketobutyrate (Saleem et al., 2021)
Leucine	9.59 ± 0.92^a^	6.48 ± 0.37^b^	4.63 ± 0.49^c^	​
Isoleucine	7.42 ± 0.39^a^	4.96 ± 0.42^b^	3.30 ± 0.24^c^	​
Asparagine	39.27 ± 2.18^a^	29.60 ± 1.53^b^	15.73 ± 0.99^c^	Hydrolytic metabolism (Nawaz et al., 1998)
Aspartic acid	2573.16 ± 89.12^a^	2372.01 ± 107.19^b^	1604.10 ± 177.94^c^	Converted to oxaloacetate (Bott, 1997)
Lysine	23.98 ± 1.73^a^	18.62 ± 1.09 ^b^	8.21 ± 0.47^c^	Degraded to glutarate (Lin et al., 2022)
Glutamine	2.90 ± 1.55^a^	1.75 ± 0.10 ^b^	1.78 ± 0.07^b^	​
Glutamic acid	212.76 ± 8.32^a^	145.93 ± 0.93 ^b^	88.56 ± 2.67^c^	Converted to α-ketoglutarate (Li et al., 2018)
Histidine	8.87 ± 0.51^a^	7.91 ± 0.25^b^	4.80 ± 0.55^c^	​
Phenylalanine	33.52 ± 1.18^a^	27.29 ± 1.30^b^	16.52 ± 1.44^c^	Converted to α-keto acids (Zhang et al., 2014)
Arginine	16.63 ± 1.21^a^	11.78 ± 0.92^b^	4.83 ± 0.58^c^	Degraded to ornithine (Lillie et al., 2024)
Tyrosine	11.26 ± 1.34^a^	10.17 ± 0.88^a^	5.83 ± 0.14^b^	Converted to α-keto acids (Drechsel et al., 1993)
Tryptophan	15.51 ± 1.08^a^	12.94 ± 1.26^b^	7.33 ± 0.38^c^	​

Different lowercase letters within the same line indicate significant differences at the p < 0.05 level.

### 3.4 Aroma components

**FIGURE 3:**
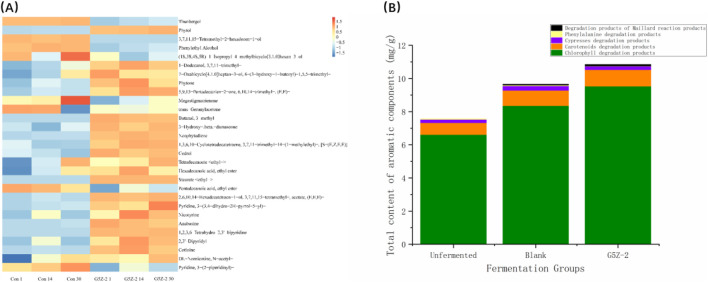
Heat map, statistics and classification of flavour substances of fermented-G5Z-2 group. **(A)** Heatmap of main aroma-contributing substances during fermentation; **(B)** different types of flavour molecules in the fermented-G5Z-2 and the fermented-control group.

The sensory characteristics of CTLs are influenced by the composition and content of aromatic compounds. During fermentation, a total of 30 aromatic compounds was identified. These compounds were categorised into eight classes, including eight alcohols, nine ketones/aldehydes, five terpenes, 6 esters, and eight alkaloids, which are frequently detected in CTLs. The total amount of aromatic compounds initially increased. Significant differences in metabolites existed throughout the fermentation cycle, and these differences gradually increased with fermentation time. For example, Maillard reaction degradation products and chlorophyll degradation products significantly increased. Overall, the fermentation process degraded macromolecules detrimental to CTLs quality and produced many compounds such as terpenes and aldehydes that determine the typical flavour of CTLs. These 30 volatile compounds can be divided into six categories based on their biosynthetic pathways, including degradation products of chlorophyll, carotenoids, phenylalanine, cembranoids, Maillard reaction products, and other products (Figure 3). Among these compounds, neophytadiene, a degradation product of chlorophyll, is the most abundant aromatic substance in cigars, accounting for over 60% of the total aromatics. Unfermented CTLs had the highest total alkaloid content; both the fermented-control and fermented-G5Z-2 group showed varying degrees of decrease in alkaloids. Excluding alkaloids, the total volatile aroma content in unfermented leaves was 7.84 mg/g. After fermentation, the fermented-control and fermented-G5Z-2 group reached 10.02 mg/g and 12.35 mg/g, respectively, representing increases of 27.8% and 57.53% compared to before fermentation. Unfermented leaves contained a significant amount of macromolecule degradation products, including 0.71 mg/g carotenoid degradation products, 0.21 mg/g cembranoid degradation products, 6.61 mg/g chlorophyll degradation products, and 0.04 mg/g phenylalanine conversion products; no Maillard reaction degradation products were detected. After fermentation, all macromolecule degradation products increased: 0.92 mg/g carotenoid degradation products, 0.27 mg/g cembranoid degradation products, 8.36 mg/g chlorophyll degradation products, 0.06 mg/g phenylalanine conversion products, and 0.04 mg/g Maillard reaction degradation products.

### 3.5 Microbial community

**FIGURE 4:**
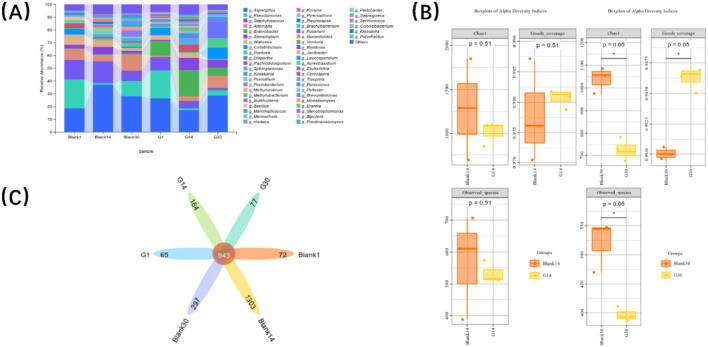
Metagenomic sequencing of G5Z-2 fermented CTLs. (**(A)** Stacked bar chart of microbial community changes in the fermented-G5Z-2 and the fermented-control group on days 1, 14, and 30; **(B)** Box plots of Chao1, Goods coverage, and observed species indices showing differences in microbial composition between the fermented-G5Z-2 and the fermented-control group on days 1 and 14; **(C)** Venn diagram of species statistics for the fermented-G5Z-2 and the fermented-control group on days 1, 14, and 30).

Metagenomic sequencing revealed the dynamic changes in the microbial community of CTLs under inoculation with G5Z-2 compared to the fermented-control group (Figure 4A). At the genus level, all samples exhibited a diverse and relatively stable core microbiota composition, but the community structure gradually changed over time. Alpha diversity index analysis (Figure 4B) showed that the differences between the inoculated and control groups were not significant in the early fermentation stage (day 1). However, in the mid to late stages (day 14 and day 30), significant differences (p < 0.05) emerged in the Chao1 index, Goods_coverage index, and Observed_species index, suggesting significant fluctuations in the richness and coverage of the leaf surface microbial community during this phase. This result indicates that although the fermentation cycle was 30 days, the critical turning point in microbial community structure likely occurred between days 14 and 30. Figure 4C shows that there were 943 shared core species among all groups. Apart from a small difference in species between the fermented-control and fermented-G5Z-2 groups on day 1, there were noticeable differences in species numbers on days 14 and 30, with the fermented-control group having far more species than the fermented-G5Z-2 group. Simultaneously, a certain number of unique species were detected at different fermentation stages, indicating that both the intervention of the inoculated strain and the time effect significantly influenced the community structure.

In all groups, the genus *Aspergillus* was almost always the most dominant microorganism in the phyllosphere microbial community, a phenomenon also reported in other literature (Rongkun et al., 2025). After introducing with G5Z-2, the proportion of *Pseudomonas* was suppressed to below 50% of its original level throughout the fermentation process compared to the fermented-control group. Not only did the species proportions change, but the Venn diagram showed that due to the introduction of G5Z-2, the alpha diversity indices of the phyllosphere microbial community on days 14 and 30 were significantly lower than those of the fermented-control group. The dominant genera during the fermented-control group process mainly included *Aspergillus*, *Staphylococcus*, *Pseudomonas*, *Alternaria*, and *Wallemia*. The dominant genera during the fermented-G5Z-2 group fermentation process mainly included *Aspergillus*, *Enterobacter*, *Pseudomonas*, *Staphylococcus*, and *Stemphylium*.

### 3.6 Metabolomic analysis

**FIGURE 5:**
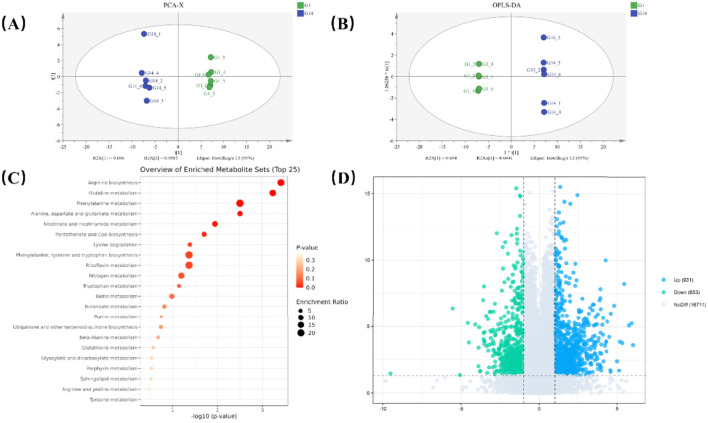
Metabolic and synthetic pathways in G5Z-2 CTLs fermentation. **(A)** PCA-X plot of metabolites from the fermentation group on day 1 and day 14; **(B)** OPLS-DA plot of metabolites from the fermentation group on day 1 and day 14; **(C)** Statistics of metabolic and synthetic pathways in the fermentation group; **(D)** Volcano plot of metabolic pathways in the fermentation group; blue represents upregulated pathways, green represents downregulated pathways, grey represents no significant difference.

Orthogonal Partial Least Squares-Discriminant Analysis (OPLS-DA) showed that Principal Component 1 (PC1) and Principal Component 2 (PC2) explained 95% of the total variance, respectively. The R2X of the OPLS-DA model was 0.939, R2Y was 0.992, and Q2 was 0.928, indicating that the OPLS-DA model displayed accurate fit to the sample data without “overfitting”. Furthermore, the closer R2 and Q2 are to 1, the more stable and reliable the model is. The above results indicate that the model fits well, has high explanatory power and strong predictive ability, and that there are significant differences between the CTLs samples. To validate the predictive and explanatory capabilities of the established OPLS-DA model, a permutation test was performed. The OPLS-DA model data underwent 200 random response permutation tests, and the permutation test results were obtained with the permutation retention rate as the abscissa and the R2 or Q2 value as the ordinate, as shown in Figure 5B. This indicates that the OPLS-DA model has strong explanatory and predictive power and can effectively distinguish these CTLs. Therefore, these results can be used for subsequent differential metabolite analysis.

Based on the PCA-X and OPLS-DA results, differential metabolites in the CTLs were further screened using the Fold Change (FC), Variable Importance in Projection (VIP), and p-value from univariate analysis. Metabolites meeting the criteria of FC > 1, VIP >1, and P < 0.05 simultaneously were considered differential metabolites, and a volcano plot was generated (Figure 5D). In the comparison between the fermented-control group and the fermented-G5Z-2 group on day 14 (G14), there were 1584 differential metabolites, of which 931 were upregulated and 653 were downregulated (Figure 5D). The upregulated metabolites indicate that 20 flavour factor metabolites were significantly higher in the fermented-control group compared to fermented-G5Z-2 group, while the downregulated metabolites indicate that eight flavour factor metabolites were significantly lower in the blank fermented group (fermented-control group). In summary, although the total fermentation cycle was 30 days, the metabolic level of the inoculated group had already significantly diverged from the early stage by day 14.

This indicates that G5Z-2 can rapidly induce a reconstruction of metabolic pathways in the CTLs fermentation system during the early stages of fermentation, including the synthesis and metabolism of various amino acids and the metabolism of nicotine, among others (specific discussion in Section 4.4).

In the correlation analysis of this study, some metabolites showed significant correlation characteristics with specific fermentation strains. The results showed that metabolites such as Cotinine, Isofraxidin, and Pilocarpine were generally highly positively correlated with multiple fermentation strains, suggesting that these compounds may accumulate accompanying the metabolic activity of specific microbial groups during fermentation. Conversely, Asparagine, Asperphenamate, and Aurantiamide acetate showed strong negative correlations with some fermentation strains, indicating that the consumption or inhibition of these metabolites may be closely related to the metabolic activities of the strains. Notably, some metabolites, such as Megastigmatrienone, showed a moderate negative correlation with *Staphylococcus nepalensis*, while *Enterobacter cloacae* showed positive correlations with some nitrogen-containing heterocyclic compounds, reflecting differences in the metabolic pathways of amino acid derivatives and aromatic compounds among different strains.

### 3.7 Sensory quality evaluation

The sensory evaluation panel from the China Tobacco Technology Innovation Centre for Cigar assessed the flavour profile of CTLs fermented with different substrates and compiled a sensory quality evaluation report. The report suggested that before and after cigar fermentation showed that pre-fermentation CTLs had strong irritation, prominent off-odours, weak aroma nuances, insufficiently mellow fragrance, and poor overall balance of the leaf. After fermentation, compared to the fermented-control group, the fermented-G5Z-2 group showed a greater reduction in leaf off-odours and irritation, enhanced mellow aroma, a cleaner and more comfortable aftertaste, and superior overall balance of the leaf. In general, compared to the natural fermentation group, the introduction of G5Z-2 significantly increased the aroma content, improved the harmony of internal chemical components, and resulted in better sensory evaluation scores.

## 4 Discussion

### 4.1 Impact on biological indicators

The results of this study indicate that G5Z-2 inoculation fermentation significantly accelerated the carbon and nitrogen metabolic transformation processes in CTLs. As fermentation time increased, the reducing sugar and total sugar content decreased faster in the fermented-G5Z-2 group, suggesting that this strain can efficiently utilise soluble sugars in the CTLs as an energy source, thereby promoting its growth and metabolism. Free sugar content is closely related to microbial metabolism, as microorganisms degrade complex carbohydrates, and microbial growth consumes nutrients in the environment, including reducing sugars (Gong et al., 2023). Additionally, during CTLs fermentation, carbonyl compounds (reducing sugars) and amino compounds undergo the Maillard reaction, consuming reducing sugars and consequently leading to a decrease in total free sugar content (Arihara et al., 2017).

Simultaneously, the more significant decrease in bioavailable nitrogen in the fermented-G5Z-2 group suggests its strong ability to degrade or transform proteins, amino acids, and other substances in the CTLs. Notably, while the free amino acid content sharply increased in the inoculated group, the total nitrogen of the CTLs decreased. This indicates that G5Z-2 not only accelerated the decomposition of nitrogenous compounds in protein and amino acid metabolism but may also have led to the release of volatile nitrogen compounds such as ammonia through deamination or nitrogen transformation pathways, resulting in nitrogen loss. On one hand, bioavailable nitrogen content is closely related to microbial metabolism and synthesis, and metabolomic predictions also revealed that G5Z-2 possesses a rich network of amino acid synthesis and metabolism. On the other hand, amino acid content is negatively correlated with CTLs sensory quality. Literature reports indicate that free amino acids have negative effects on sensory quality indicators such as aroma quality, off-odours, irritation, aftertaste, dryness, fineness, softness, roundness, total sensory score, and the appearance quality indicator leaf structure (Guo et al., 2018). Introducing microbial fermentation can reduce the irritation of CTLs by decreasing the total amino acid content.

To identify the intrinsic driving factors of quality formation during the process, we measured the specific activities of carbon-nitrogen metabolism enzymes (AL, PRO), enzymatic browning-related enzymes (POD and PPO), and polyphenol synthesis enzyme (LPO) during the process. We found that enzyme activities fluctuated stage-wise and were relatively stable during the fermentation stage. Compared to the fermented-control group, the activities of LPO, PPO, and POD increased significantly during the reaction fermentation stage, with LPO activity increasing from 21 U/g in fresh leaves to a peak of 79 U/g; AL activity peaked at 5.23 U/g in the initial stage and then gradually decreased to 1.60 U/g by the end of the fermentation stage. Although both fermented-control and fermented-G5Z-2 groups approached the second highest level at fifth day separately, it was assumed as a limited bacterial metabolic strategy adjustment that does not affect the overall trend, both in terms of microbial and metabolic outcomes. The decline in amylase activity in the late fermentation stage may be due to polysaccharide substrate depletion and decreased microbial activity.

Enzyme activity assays further support this speculation: polyphenol oxidase, peroxidase, and lipoxygenase activities were all significantly enhanced in the inoculated group, indicating that G5Z-2 can intensify oxidative degradation reactions, accelerating the breakdown and transformation of substrates both inside and outside cells. Changes in free amino acids and protease activity, along with microbial growth, are consequences of synergistic effects during fermentation (Ma et al., 2024). Proteases during fermentation can reduce protein content, lessen foul off-odours and bitter tastes, increase sweet aroma and improve aftertaste, and enhance the fragrance of CTLs (Zhang et al., 2024). During fermentation, protease activity showed a fluctuating trend, reaching its highest peak on day 3, possibly due to the addition of exogenous microorganisms causing increased protease activity. fermented-G5Z-2 group reached its lowest activity on day 7, while the fermented-control group reached its lowest value on day 5, potentially because exogenous microorganisms prolonged the expression of protease activity. All groups showed a stable trend in the later stages of fermentation. In summary, G5Z-2, through its active carbon-nitrogen metabolism and oxidase system, promoted sugar consumption, nitrogen transformation, and metabolite accumulation during CTLs fermentation, consequently leading to reduced total nitrogen levels in the leaves post-fermentation.

### 4.2 Impact on flavour

From the perspective of aroma substance formation, many macromolecular degradation processes, such as terpene degradation, higher fatty acid degradation, and polyphenol degradation, involve key roles for peroxidase, lipoxygenase, and polyphenol oxidase (Wu et al., 2021). We found that the fermented-G5Z-2 group had higher lipoxygenase, polyphenol oxidase, and peroxidase activities in the mid to late stages of fermentation, which may have synergistically promoted the degradation of carotenoids, higher fatty acids, and polyphenols, thereby contributing to higher levels of carotenoid degradation products, cembranoid degradation products, and Maillard reaction precursors. Lipoxygenase is associated with the degradation of fatty acids and carotenoids in CTLs (Li et al., 2021; Lyu et al., 2021), so lipoxygenase plays an important role in enhancing the aroma and reducing irritation of CTLs during aging. Lipoxygenase activity generally showed a gradually increasing trend, and the lipoxygenase activity in the fermented-G5Z-2 group was greater than that in the control group for the first 21 days of fermentation. Polyphenol oxidase and peroxidase are important enzymes in the degradation or transformation processes of phenolic substances (Qiu et al., 2003; Yong et al., 2023). Although polyphenol oxidase activity showed some fluctuation during fermentation, the activity in the fermented-G5Z-2 group was consistently greater than that in the control group throughout the entire process. Peroxidase activity remained stable for the first 14 days and then showed an increasing trend.

Furthermore, this study examined amylase activity from the perspective of polysaccharide degradation. Amylase activity increased in the mid-fermentation stage and then gradually decreased. With the growth and reproduction of exogenous active microorganisms, amylase activity showed an increasing trend in the later stage. The decline in amylase activity in the late fermentation stage may be due to polysaccharide substrate depletion and decreased microbial activity. We speculate that the rise in amylase activity in the fermented-G5Z-2 group in the later stage may be related to enhanced utilisation of starch by the G5Z-2 strain or specific microorganisms it induced during the later phase. The sequential peaking of various enzymes during fermentation reflects the biosynthetic and catabolic preferences of the microbial community at different fermentation stages and also indicating the different timings for the generation of flavour-related metabolites.

### 4.3 Microbial changes

Research focusing on the relationship between fermentation outcomes and microbial abundance has concluded that highly abundant dominant microorganisms (relative abundance >1%) play an important role in the fermentation process (Wolfe et al., 2014). Combining the microbial characterisation results from this CTLs fermentation, we propose that the succession and metabolic activities of the microbial community are key drivers of flavour formation during CTLs fermentation. The genus *Aspergillus*, as a core functional fungus, secretes amylases, proteases, and cellulases to degrade CTLs polysaccharides, proteins, and cellulose, generating reducing sugars and free amino acids, that provide precursors for the Maillard reaction. Simultaneously, it promotes carotenoid degradation to form aroma-contributing components like megastigmatrienone, imparting sweet and fruity notes to the cigar. The genus *Staphylococcus* dominates nitrogen compound metabolism, effectively degrading proteins and alkaloids, reducing leaf irritation, and improving smoke smoothness. The genus *Pseudomonas*, through lipoxygenase catalysis, promotes fatty acid oxidation and polyphenol degradation, reducing green and off-odours and generating aldehyde and ketone aromas, but its abundance is easily suppressed by exogenous bacterial influence (Zhang et al., 2025). The genus *Enterobacter*, as an exogenous beneficial bacterium, possesses GH1 family glycoside hydrolases that efficiently hydrolyze glycosidic bonds to release bound aroma precursors, significantly increasing the content of solanone, megastigmatrienone, and other aroma compounds. At the molecular and ecological levels, *Enterobacter* G5Z-2 may reshape the microbial community structure by outcompeting native flora such as *Pseudomonas* via niche exclusion and nutrient competition, thereby altering the overall metabolic flux. Additionally, the genus *Alternaria* may participate in cellulose degradation; the genus *Wallemia* may regulate organic acid metabolism in low-moisture environments. Although the function of the genus *Stemphylium* remains to be clarified, the synergistic effect of the microbial community resulted in a significant improvement in CTLs flavour.

The inoculation of G5Z-2 not only maintained a certain stability of the core microbial community during fermentation but also significantly influenced the trend of diversity changes on the CTLs surface. Particularly between days 14 and 30 of fermentation, significant differences in multiple alpha diversity indices indicate that this stage may be a critical period for community succession. This aligns with the metabolomics analysis results, which showed significant metabolic differences already present at day 14. We therefore propose a mechanistic hypothesis: G5Z-2 colonization drives metabolic pathway shifts at the molecular level by modulating microbial community structure and dominant population composition. Specifically, G5Z-2 may upregulate glycoside hydrolysis, redox reactions, and aroma precursor release while suppressing undesirable microbial metabolism, enabling a faster transition of CTLs from the initial state to a stable fermented state (Yang T et al., 2025). This finding suggests that inoculating beneficial strains during CTLs fermentation can accelerate community dynamic adjustments, causing key quality-related metabolic changes to occur earlier, thus potentially shortening the required fermentation time and optimising the final quality.

### 4.4 Changes in metabolic pathways

G5Z-2 inoculation can significantly alter the metabolic state of CTLs in a short period, particularly eliciting a rapid response in amino acid and nitrogen-containing compound metabolism. This is likely closely related to the efficient utilisation of nitrogen sources by *Enterobacter* in the CTLs matrix, promoting protein degradation and amino acid metabolism, thereby providing precursor substances for subsequent Maillard reactions and the generation of pyrazines and pyrroles (Chen et al., 2019). Furthermore, the enrichment of pathways such as nicotinate/nicotinamide metabolism also suggests that G5Z-2 may affect energy metabolism and coenzyme regulation, metabolically degrading some nicotine, thus indirectly promoting the formation of flavour-related substances. Notably, metabolomic data indicate that significant metabolic restructuring occurring by day 14. This implies that key metabolic regulation and flavour substance transformation may be largely completed in the early stages of fermentation. This finding provides an experimental basis for optimising fermentation processes, shortening fermentation cycles, and enhancing the quality of CTLs.

The metabolic profiles were significantly separated. Both PCA and OPLS-DA analyses showed complete separation of the two groups along the dominant metabolic dimension (PC1, explaining 69.6% of variation): the G-1 group clustered in the positive region (t [1]≈7), the G-14 group was located in the negative region (t [1] ≈ −7.5), and the G-14 group exhibited higher metabolic heterogeneity. Among the pathways, arginine biosynthesis (p < 0.001) and phenylalanine metabolism (p < 0.001) were significantly enriched, suggesting enhanced oxidative stress defense and significant differences in neuroendocrine regulation compared to other groups; disturbance in histidine metabolism (p < 0.001) indicated activated responses.

Phenylalanine-tyrosine-tryptophan synthesis and riboflavin metabolism were moderately enriched (larger p-values), reflecting imbalances in aromatic amino acid homeostasis and potential impairment in mitochondrial energy metabolism. The G-14 group exhibited stress-adaptive remodeling (upregulated arginine/histidine metabolism) and compensatory energy metabolism (fluctuations in riboflavin-related pathways).

KEGG pathway topology analysis is a method primarily based on the structure of each cyclic reaction and the relative positions of biomolecules, using weighted scores to assess the relative importance of metabolites or biomolecules to a pathway (Li et al., 2020). Using the pathway analysis function of MetaboAnalyst, KEGG pathway enrichment analysis was performed on differential metabolites. The top 25 most significant pathways were screened, as shown in Figure 5C. In Figure 5C, each bubble represents a KEGG pathway. The x-axis represents the impact value of the metabolites in the pathway, while the y-axis represents the significance of metabolite enrichment in the pathway [-log10(P-value)]. The node colour ranges from yellow to red; the redder the color, the smaller the P-value, and the larger the node radius, the larger the impact value. Points farther from the axes, with deeper colours and larger sizes, indicate that the metabolic pathway is more significantly affected. In the comparison between the fermented-control group and the fermented-G5Z-2 group, a total of 18,295 metabolites were annotated to Kyoto Encyclopedia of Genes and Genomes (KEGG) pathways, involving 116 metabolic pathways. The three metabolic pathways with the highest P-values were alanine, aspartate, and glutamate metabolism; nucleotide metabolism; and flavonoid degradation. The largest number of successfully annotated metabolites was observed in the biosynthesis of secondary metabolite pathways. Metabolomic analysis results showed that G5Z-2 inoculation fermentation significantly altered the metabolic profile characteristics of CTLs (Figures 5A–D). Metabolic pathway enrichment analysis (Figure 5C) indicated that differential metabolites were primarily concentrated in multiple amino acid and nitrogen metabolism pathways such as arginine and histidine metabolism, phenylalanine metabolism, glutamate and aspartate metabolism, and nicotinate and nicotinamide metabolism, suggesting significant regulation of nitrogen metabolism during fermentation. Principal Component Analysis (PCA) is shown in Figure 5A, where the two principal components cumulatively contributed 95% of the variance. Analysis of the CTLs samples revealed a lack of clear distinction between samples based on their characteristic substances, with significant clustering observed among samples. However, significant differences were observed between different sample groups. Samples from fermentation day 1 (G1) and day 14 (G14) were clearly separated, indicating significant differences in their metabolic profiles.

**FIGURE 6:**
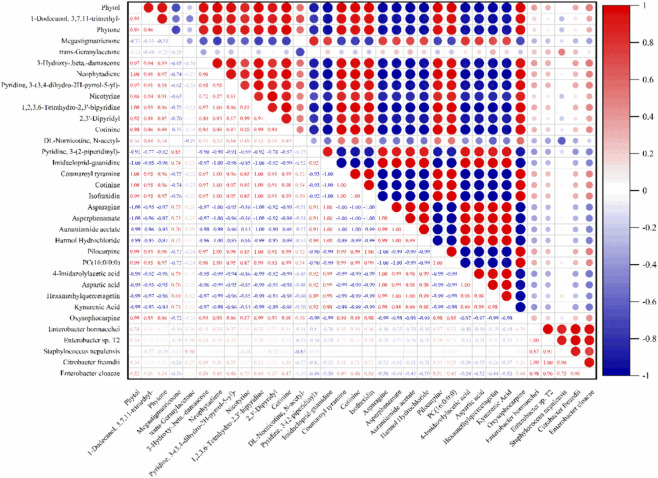
Correlation analysis of flavour substances, dominant phyllosphere strains, and strain metabolites in G5Z-2 fermentation. The diameter of the red circles and the associated red numerical values indicate the strength of the positive correlation between the metabolites and/or microbial species corresponding to each row and column. Conversely, the diameter of the blue circles and the accompanying blue numerical values represent the magnitude of the negative correlation between the respective row and column variables.

To further explore the potential connections between bacteria and metabolites, correlation analysis was performed between major metabolites and dominant bacterial genera during fermentation (Figure 6). The results showed significant positive and negative correlation patterns both among different metabolites and between metabolites and bacterial species. Pigment degradation products such as Phytol, Phytene, Megastigmatrienone, and β-Damascenone were highly positively correlated with each other, suggesting they may originate from the same or similar metabolic pathways. Simultaneously, alkaloids and their derivatives such as Nicotine, Cotinine, and Neophytadiene also exhibited strong positive correlations among themselves, reflecting a coordinated trend in their metabolic processes. Conversely, some amino acids (e.g., Asparagine, Aspartic acid) showed significant negative correlations with typical aroma substances (e.g., β-Damascenone), indicating that amino acids may be consumed as precursor substances, thereby promoting the generation of aroma components. Regarding the relationship between bacteria and metabolites, *E. hormaechei* and *Enterobacter* sp. T2 showed positive correlations with various volatile degradation products (e.g., Neophytadiene, Nicotine derivatives), suggesting they may play key roles in promoting the formation of flavour substances. *Citrobacter freundii* was positively correlated with various pyridine and aromatic compounds, indicating that this strain is more inclined to participate in the synthesis of nitrogen-containing flavour substances. Relatively, *S. nepalensis* and *E. cloacae* showed negative correlations with some metabolites, implying that their metabolic activities might promote the decomposition and consumption of certain substances. In summary, this correlation analysis revealed complex interactions between bacteria and metabolites during fermentation: pigment degradation products and alkaloids showed coordinated accumulation; amino acids and aroma components exhibited a substrate-product consumption relationship; and different bacterial species showed functional differentiation in their metabolic directions. The results indicate that *Enterobacter* and *Citrobacter* strains play important roles in the formation of flavour substances during CTLsfermentation, potentially regulating the final aroma characteristics by promoting the activation of specific metabolic pathways.

Detection results of flavour substances in CTLs indicated that their total content was significantly higher after inoculation with G5Z-2 fermentation compared to Blank1 and Blank30 (Figure 3). Among them, chlorophyll degradation products and carotenoid degradation products were the main components, with the highest content proportion, and they gradually increased with fermentation treatment. The contents of phenylalanine degradation products, cembranoid degradation products, and Maillard reaction products were relatively low but increased in the fermented-G5Z-2 group compared to the fermented-control. The results indicate that bacterial fermentation not only promoted the decomposition and transformation of pigment compounds but also drove the occurrence of amino acid and sugar-related reactions, thereby generating a more diverse range of flavour substances. Existing research shows that chlorophyll and carotenoid degradation products can impart unique aromatic characteristics to CTLs, while amino acids and Maillard reaction products play important roles in improving mellow sensation and aroma harmony. Therefore, G5Z-2 inoculation fermentation, by enhancing the total amount and diversity of flavour substances, contributes to improving the aroma quality and overall sensory characteristics of CTLs.

Alanine conversion products can impart floral notes to CTLs and increase the cleanliness of the aftertaste; carotenoid degradation products can reduce tobacco irritation. Cembranoid degradation products possess a fresh aroma and can enhance the smoothness of the smoke (Banožić et al., 2021). Maillard reaction products can impart nutty and other aromas to tobacco, making its aroma nuances more diverse (Yong et al., 2023). The content of these aroma components is closely and positively correlated with cigar quality. Based on metabolic pathway analysis, it was found that the introduction of G5Z-2 lead to changes in carbon and nitrogen metabolic pathways, resulting in significant changes in total sugar, reducing sugar, protein, amino acids, nicotine, and volatile components. To more comprehensively analyse the synthesis mechanisms of volatile flavour compounds, metabolic pathways were visually presented based on the KEGG database (Figure 6). Tryptophan, tyrosine, and phenylalanine are important precursor substances. During fermentation, tryptophan and phenylalanine showed a gradual decreasing trend, while tyrosine first increased and then decreased, leading to the production of substances such as *N*-Acetylserotonin, picolinic acid, 4-Hydroxyphenylacetyl glycine, as well as succinate and 4-hydroxyvalerate.

## 5 Conclusion

This study demonstrates that introducing *Enterobacter hoffmannii* G5Z-2, a functional strain derived from Citrus reticulata ‘Chenpi’, can effectively enhance the fermentation quality and aroma profile of CTLs. Multi-omics analyses revealed that G5Z-2 colonisation reduced microbial alpha diversity and suppressed native *Pseudomonas*, while promoting the dominance of core functional fungi such as *Aspergillus*. Through niche competition and metabolic cooperation with taxa such as *Staphylococcus*, G5Z-2 reinforced microbial functional partitioning and intensified key enzyme activities (lipoxygenase, polyphenol oxidase, peroxidase), facilitating the degradation of carotenoids, cembranoids, and polyphenols.

Metabolomic profiling confirmed the enrichment of arginine biosynthesis and phenylalanine metabolism pathways, supplying critical precursors for the Maillard reaction and resulting in a marked increase in volatile aroma compounds. Post-fermentation CTLs exhibited elevated levels of key aroma constituents—Maillard derivatives, phenylalanine conversion products, and carotenoid degradation products—collectively imparting nuanced nutty, floral, and smooth sensory attributes. These findings provide a mechanistic framework for the targeted application of plant-derived functional microorganisms in cigar fermentation and support the potential for bio-enhanced development of distinctive Chinese-style cigar flavours.

## Data Availability

The metagenomic and metabolomic data presented in the study are deposited in the NCBI repository, accession number PRJNA1453761: https://www.ncbi.nlm.nih.gov/bioproject/PRJNA1453761.
